# The Development of a Monoclonal Antibody Recognizing the *Drosophila melanogaster* Phosphorylated Histone H2A Variant (γ-H2AV)

**DOI:** 10.1534/g3.113.006833

**Published:** 2013-09-01

**Authors:** Cathleen M. Lake, Julie Korda Holsclaw, Stephanie P. Bellendir, Jeff Sekelsky, R. Scott Hawley

**Affiliations:** * Stowers Institute for Medical Research, Kansas City, Missouri 64110; † Curriculum in Genetics and Molecular Biology, University of North Carolina, Chapel Hill, North Carolina 27599; ‡ Department of Molecular and Integrative Physiology, University of Kansas Medical Center, Kansas City, Kansas 66160

**Keywords:** γ-H2AV, H2AX, double-strand break, DNA repair, meiosis

## Abstract

The recognition of DNA double-strand breaks (DSBs) using a phospho-specific antibody to the histone 2A variant has become the gold standard assay for DNA damage detection. Here we report on the development of the first monoclonal antibody to the phospho-specific form of *Drosophila* H2AV and characterize the specificity of this antibody to programmed DSBs in oocytes and rereplication sites in endocycling cells by immunofluorescence assays and to DSBs resulting from irradiation in both cell culture and whole tissue by Western blot assays. These studies show that the antibody derived in the study is highly specific for this modification that occurs at DSB sites, and therefore will be a new useful tool within the *Drosophila* community for the study of DNA damage response, DSB repair, meiotic recombination and chemical agents that cause DNA damage.

The ability of a cell to recognize DNA damage and repair is an essential process for cell survival and genetic stability. Within a cell, DNA damage can occur by intrinsic insults like metabolic stress or programmed DNA double-strand breaks (DSBs) in meiocytes or during immune system development. DSBs can also be induced by extrinsic insults such as chemical and environmental factors. Regardless of which type of mutagenic event caused the DSB, the cell must be able to recognize the lesion and repair it before continuing on with the cell cycle. Failure to repair the DSBs could result in chromosomal instability, genomic aberrations, and segregation defects in both meiosis and mitosis, all of which could promote tumorigenesis and/or cell death in mitotic cells or pachytene arrest in meiosis (reviewed in Jackson and Bartek 2009; MacQueen and Hochwagen 2011).

DSBs trigger activation of the DNA damage response pathway and DNA repair occurs through an elaborate mechanism involving DNA damage checkpoints that prevent the cell cycle from proceeding until the lesion is repaired (reviewed in Polo and Jackson 2011). One of the earliest events after DSBs form, regardless of whether it was a physical, biological, or chemical event, is the activation of protein kinases (including ATM, ATR, and DNA-PK) that rapidly phosphorylate the C-terminal tail of the histone 2A variant (reviewed in Talbert and Henikoff 2010). This phosphorylation, which occurs at the DSB site, is an evolutionarily conserved response throughout multiple eukaryotic systems (Rogakou *et al.* 1999). In *Drosophila* the phosphorylation occurs on the histone 2A variant (H2AV) (Madigan *et al.* 2002; Rogakou *et al.* 1999) and in mammals is named H2AX (Rogakou *et al.* 1998).

Conserved residues/motifs located in this C-terminal tail are found in most eukaryotic H2AX variants (Redon *et al.* 2002). Specifically, the SQ phosphorylation motif located precisely four amino acids from the end of the protein, is known to be posttranslationally modified in response to DSBs (Redon *et al.* 2002; Rogakou *et al.* 1998, 1999). The phosphorylated form of H2A variants is denoted γ-H2AV in flies and γ-H2AX in mammals and occurs at conserved serines S137 or S139 in flies and mammals, respectively (Madigan *et al.* 2002; Rogakou *et al.* 1998, 1999). Although this modification initially occurs at the DSB site itself, the signal can be extended to megabases of DNA adjacent to the DSB site in mammals (Rogakou *et al.* 1999) and up to 50 kb of DNA in yeast (Downs *et al.* 2004; Shroff *et al.* 2004).

Because the phosphorylation of serine is an evolutionarily conserved response and the fact it is a rapid event, detecting γ-H2AX is considered to be the hallmark assay in both mitotic and meiotic systems for DSB recognition. In addition, studies have shown that the number of DSBs correlates with the number of γ-H2AX foci (Rogakou *et al.* 1999). Although polyclonal antibodies to human γ-H2AX can detect *Drosophila* γ-H2AV in Western blots (Rogakou *et al.* 1999), studies have shown that these antibodies lack specificity in meiotic *Drosophila* tissue by immunocytological assays (Jang *et al.* 2003; Mehrotra and McKim 2006). More recently, polyclonal rabbit antibodies have been developed against H2AV and γ-H2AV, and these studies have lead to insights in chromosomal H2AV distribution throughout the genome in both polytene and diploid chromosomes (Leach *et al.* 2000), as well as provided us with the detailed analysis of the timing of meiotic DSB formation and repair (Mehrotra and McKim 2006). However, we wanted to produce a monoclonal antibody to γ-H2AV because monoclonal antibodies often have low background, are highly specific to one epitope and can be produced in a homogeneous population in large quantities. Here we describe the first monoclonal antibody against phosphorylated *Drosophila* histone 2A variant (γ-H2AV) and characterize the specificity and use of this antibody by immunocytological assays in both meiotic and somatic tissue and on Western blot assays.

## Materials and Methods

### Antibody production

**Figure 1:**
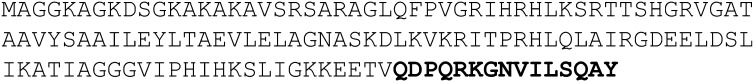
Sequence of the *Drosophila* H2AV protein. The amino acid sequence used to generate the γ-H2AV antibody is shown in bold (FlyBase). The serine within this sequence is phosphorylated in response to DSB formation (Madigan *et al.* 2002). Sequence alignment of the C-terminal tail of the *Drosophila* H2AV to other eukaryotic H2AX can be found in Redon *et al.* (2002).

A phosphorylated peptide QDPQRKGNVILSQAY, which corresponds to the last 15 amino acid residues of the H2AV protein, was synthesized by GenScript with a phosphate added to the serine (Figure 1). The peptide was conjugated to Keyhole Limpet Hemocyanin via an N-terminal cysteine added to the peptide. Monoclonal antibody production was performed at the University of North Carolina Immunology Core Facility. Four mice were immunized three times over 9 wk with 50 μg of peptide per mouse per intraperitoneal injection. After two injections, mice were bled to test for response in both an enzyme-linked immunosorbent assay to phosphorylated and nonphosphorylated peptide and by immunocytological assays. One mouse was chosen for fusion. The most productive clone, which was determined by immunocytological assays, was chosen and subsequently underwent two rounds of subcloning before expansion and Protein A purification. The monoclonal antibody derived in this study has the isotype IgG2b-kappa (by Isostrip Kit, Roche Applied Science). It is the intent of the authors to deposit the hybridoma clone used in this study into a hybridoma bank for easy distribution.

### *Drosophila* stocks used in the study

All stocks were maintained on standard food at 25°. The wild-type stock used for immunological analysis was *y^1^ w^1118^ FRT19A*/ *y+Y*, derived from Bloomington Stock Center 1744 *y^1^ w^1118^ P{ry^+t7.2^ = neoFRT}19A* (Collins *et al.* 2012). The wild-type stock used for Western blot assay was *w^1118^*. Other stocks used include *y*; *mei-W68^4572^* /*CyO* (Mehrotra and McKim 2006), *net dp ho b mei-W68^4572^*, *okra^AA^ cn bw*/*CyO*, *okra^RU^ cn bw*/*CyO* (Ghabrial *et al.* 1998), and *his2AV^810^/TM3*, *Ser P{w^+^*, *Act*::*GFP}*, derived from Bloomington Stock Center 9264 (van Daal and Elgin 1992). *mei-W68* refers to the genotype *mei-W68^4572^/ net dp ho b mei-W68^4572^* and *okra* refers to the genotype *okra^AA^ cn bw*/*okra^RU^ cn bw*.

### Immunological assay

For ovarian tissue, early egg chambers were collected, fixed and immunostained as previously described (Lake *et al.* 2011). For the initial analysis of hybridoma clones, supernatant from each clone was used neat. Supernatant from the expansion line was used at 1:500−1:1000 dilution and Protein A purified antibody at a concentration of 1 mg/mL was used at a dilution of 1:2000−1:4000. From the expansion line, both the supernatant and purified antibody at the aforementioned dilutions produced similar results in this assay. Guinea pig anti-C(3)G antibody, used as a marker for synaptonemal complex, was used at a dilution of 1:500 (Page and Hawley 2001). Secondary goat anti-mouse or guinea pig IgG (H&L) Alexa-488 or Alexa-555 conjugated antibodies (Molecular Probes) were used at 1:500.

For S2 cells, cells were plated at 1 − 10^6^ cells/mL on concanavalin A−treated coverslips as described in (Rogers and Rogers 2008). After 1-hr attachment, cells were irradiated with 1000 rads from a ^137^Cs source (Gammacell GG10) then allowed to recover for 15 min. Staining was modified from (White *et al.* 2011). In summary, cells were fixed for 10 min in 10% formaldehyde, washed twice with 1× phosphate-buffered saline (PBS), and permeabilized for 15 min with 0.2% Tween in 1× PBS. After one wash with 1× PBS, samples were blocked for 1 hr with 2% NGS in 1× PBS and incubated overnight at 4° with Protein A purified mouse anti-γ-H2AV (1:2500). The primary antibody was visualized with goat antimouse IgG-Cy3 (1:2500, Jackson ImmunoResearch Laboratories, Inc.). DNA was detected by staining with DAPI (1:1000, Molecular Probes, Inc.) for 1 min at room temperature.

### Microscopy

Imaging of ovarian tissue was done using a DeltaVision microscopy system (Applied Precision, Issaquah, WA) equipped with an Olympus 1× 70 inverted microscope and high-resolution charge-coupled device camera. The images were deconvolved using the SoftWoRx v.5.5 software (Applied Precision) and projected with multiple stacks. Individual foci were counted using Imaris version 7.0.0 software (Bitplane, Zurich, Switzerland). Imaging of S2 cells was done with a Leica TCS SP5 confocal microscope and analyzed with the Leica Microsystems LAS AF software.

### *Drosophila* stocks and sample prep

Larvae homozygous for the amorphic allele *H2AV^810^* were obtained by crossing males and females with the genotype *His2Av^810^/TM3*, *Ser P{w^+^*, *Act*::*GFP}* then selecting larvae not expressing GFP, using a fluorescent dissecting microscope. Control flies and larvae were homozygous *w^1118^*. To obtain larval samples flies were crossed, allowed to mate for 3 d (brood 1) then transferred to a new vial for another 3 d (brood 2) before being discarded. The larvae from each vial were collected separately to a volume of 100 μL. To obtain fly samples, 40 flies were collected from the same vial and separated evenly into two sample groups. Brood 1 larvae and 20 flies were subjected to 1000 rads gamma radiation from a ^137^Cs source (Gammacell GG10) separately, whereas brood 2 and the remaining 20 flies received no treatment. After a 5-min recovery, all larvae and flies were frozen at −80° and ground in sodium dodecyl sulfate-polyacrylamide gel electrophoresis (SDS-PAGE) sample buffer.

### Cell culture

S2 cells were maintained in SF-900 media with 1:1000 dilution pen/strep antibiotic at 25°. Three milliliters of S2 cells were plated onto two 60-mm × 15-mm plates at a concentration of 2.2 × 10^6^ cells/mL and allowed to grow for 2 d. One plate was exposed to 1000 rads ionizing radiation (IR) as described previously, whereas the other plate received no treatment. After a 10-min recovery, the cells were washed from the plate and spun down at 2400 × g for 5 min. The supernatant was removed, and the cells were lysed by pipetting vigorously in SDS-PAGE sample buffer.

### Western blots

All samples were run on a 15% SDS-PAGE gel at 125 V for 1.5 hr. Volumes loaded were adjusted to standardize protein concentration based on loading control (α-tubulin) and estimated at approximately one fly or three larvae per lane. Proteins were transferred for one hour at 160 mA. The membrane was cut at the 31-kDa marker and the upper portion was incubated with 1:20,000 dilution purified mouse anti-α-tubulin antibody while the lower portion was incubated with 1:20,000 dilution Protein A purified mouse anti-γ-H2AV antibody. Both membranes were incubated with 1:20,000 dilution goat α-mouse secondary antibody (Santa Cruz Biotechnology; sc-2055, lot J2212). The membranes were incubated with ECL (Thermo Scientific 34080, lot NC170863) before exposure to film.

## Results and Discussion

### Generation of a monoclonal γ-H2AV antibody

A phospho-peptide to the C-terminal 15 amino acid residues of *Drosophila* H2AV (Figure 1) was used to generate a monoclonal antibody by standard methods (see *Materials and Methods*). Hybridoma clones were screened by enzyme-linked immunosorbent assay for specificity to the phospho-specific form of H2AV and also by immunocytological assays to address specificity to programmed meiotic DSBs by screening meiotic tissue from DSB repair-deficient females (data not shown). One hybridoma line was chosen for further expansion and analysis.

### Specificity of γ-H2AV monoclonal antibody in meiotic tissue

*Drosophila* females are an ideal system to test the specificity of an antibody to γ-H2AV because the ovaries are arranged according to developmental age and because we can easily control the presence or absence of DSBs by using mutants that block their occurrence or repair. Therefore, within one ovariole we can visualize the induction of programmed DSBs that occur in pro-oocytes in early prophase. In the more mature oocytes we can see that the process of DNA repair is underway or complete as indicated by the removal of the γ-H2AV modification.

**Figure 2:**
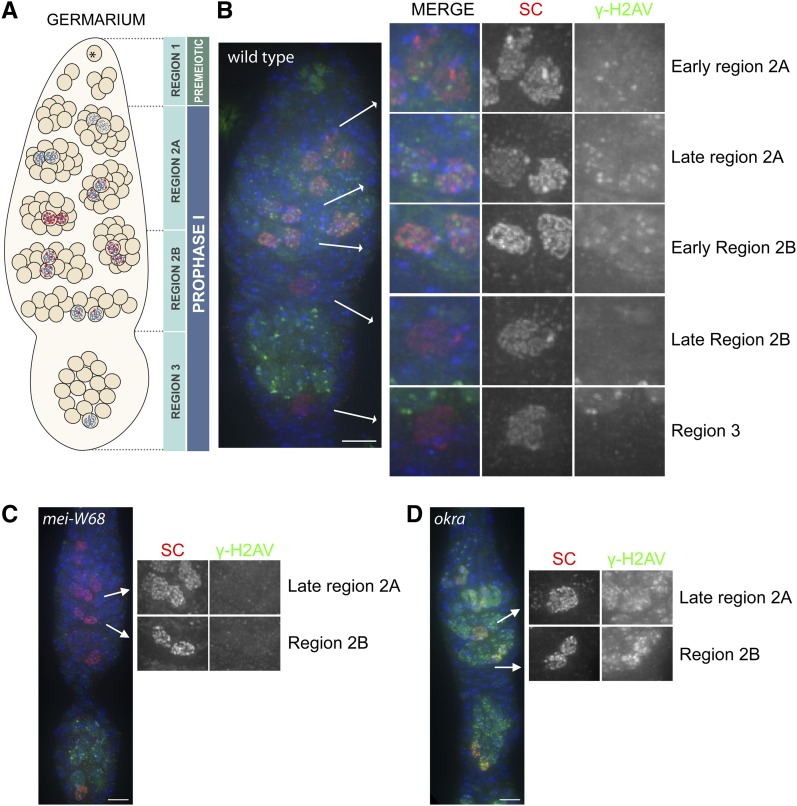
Timing of DSB formation and repair as assayed by γ-H2AV staining in wild-type and mutant oocytes. (A) A schematic of the events in meiotic prophase in the *Drosophila* ovary showing the timing of DSB formation and repair in the germarium. In region 1 the germline stem cell (*) divides to produce a germline stem cell and a cystoblast. The cystoblast then undergoes four rounds of incomplete mitotic divisions to produce a 16-cell interconnected cyst. Meiosis initiates within several cells of the 16-cell cyst and synaptonemal complex (SC) (blue ribbon) can be visualized at this stage (region 2A). Programmed meiotic DSBs are induced (red dots) after SC forms in early region 2A. As the cyst matures and moves more posterior in the germarium the number of γ-H2AV foci within the pro-oocytes (marked by SC), and therefore the number of DSBs, decreases throughout region 2B (early/mid-pachytene). By region 3 (mid-pachytene) there are no foci. The removal of the γ-H2AV signal indicates that DSB repair is underway and/or complete. (B−D) Immunocytological analysis of oocytes from wild-type (B), DSB deficient *mei-W68* (C), and DSB repair−deficient *okra* (d) females showing the timing of DSB formation as marked by Protein A purified mouse anti-γ-H2AV (green) and repair (the removal of γ-H2AV signal) in pro-oocytes (identified with SC by anti-C3G in red) and DAPI (blue). Selected pro-oocyte nuclei in each stage are shown in 2X magnification to the far right. The γ-H2AV signal seen in the 15 surrounding nurse cells that surround the oocyte in the region 3 cyst are caused by the initiation of the endoreduplication cycle. In 33% of wild-type germarium (n = 12) one to a few nuclei in the tip of germarium were positive for γ-H2AV staining (seen in B). The origin of these nuclei is unknown at this time. Scale bar: 5 µm.

As mentioned previously, studies using a polyclonal γ-H2AV antibody have precisely detailed the timing and repair of meiotically induced DSBs in *Drosophila* females (Mehrotra and McKim 2006). Therefore, we chose to first analyze the specificity of the monoclonal γ-H2AV in an immunocytological assay by comparing the timing of DSB formation and repair as indicated by the detection and removal of γ-H2AV signal during prophase (overview shown in Figure 2A) to the previously published data. To summarize, DSBs are induced in early region 2A (early pachytene), after synaptonemal complex formation. In region 2B (early-mid pachytene), the number of foci is reduced from that seen in region 2A. Once the oocyte reaches the end of the germarium, region 3 (mid-pachytene), the phosphorylated H2AV mark is completely removed.

The staining pattern of the antibody derived in this study shows a nearly identical pattern to that observed with the existing polyclonal antibody in the initial studies (Figure 2B) (Mehrotra and McKim 2006). In early region 2A when DSBs are first apparent using anti-γ-H2AV, the average number of foci per pro-oocyte was 5.5 (N = 17). In late region 2A, the peak of γ-H2AV staining, the average number of foci per pro-oocyte was 10.8 (N = 23). The number of foci then decreased throughout region 2B with an average of 3.8 foci per pro-oocyte, until virtually all the γ-H2AV modification had been removed by region 3 (0.25 foci per oocyte, N = 8). We also detected γ-H2AV staining in region 3 nurse cells, the remaining 15 cells within the 16-cell cyst. This staining is not attributable to the preprogrammed meiotically induced DSBs seen in early prophase but rather to the first endocycle S phase, which occurs only in the nurse cells (Lilly and Spradling 1996; Hong *et al.* 2007; Mehrotra *et al.* 2008). This finding indicates not only that the γ-H2AV antibody is recognizing the phosphorylation of H2AV during prophase in response to meiotically induced DSBs but also those formed during endocycle replication.

In addition, we tested the specificity and background of this antibody on meiotic tissue from mutant *Drosophila* females that were either defective in DSB formation or defective in the ability to repair meiotically induced DSBs. In *Drosophila*, DSBs are created by the topoisomerase II-like Mei-W68 (Keeney *et al.* 1997; McKim and Hayashi-Hagihara 1998). Our immunocytological analysis, in which we used the γ-H2AV monoclonal antibody on *mei-W68* mutant ovaries, indicated that the antibody specifically recognized the programmed DSBs in wild-type ovaries during prophase because no signal or discrete foci were detected at this stage compared with control ovaries (Figure 2C, compare to Figure 2B). Similar to that seen in wild-type germarium, in *mei-W68* mutant ovaries, anti-γ-H2AV detected DSBs that result from entry into the endocycles in region 3 nurse cells. Another meiotic mutant, *mei-P22*, whose function is required for Mei-W68 to exert its activity (Liu *et al.* 2002), showed similar results to *mei-W68* (data not shown).

We also analyzed the γ-H2AV staining pattern on ovaries defective in the ability to repair DSBs. Okra, the Rad54 ortholog, was required for repair of meiotically induced DSBs (Kooistra *et al.* 1997; Ghabrial *et al.* 1998); therefore, in its absence the phosphorylation of H2AV was not removed in the pro-oocytes and γ-H2AV foci were still visible in mid-pachytene oocytes (region3) (Figure 2D) (Mehrotra and McKim 2006). We find that the number of γ-H2AV foci in region 2B and region 3 oocytes using this antibody was consistent with what has been previously published for DSB repair deficient mutants (19.3 foci average in region 2B, N = 10 and 23.6 foci average in region 3, N = 10) (Jang *et al.* 2003; Mehrotra and McKim 2006; Klovstad *et al.* 2008). As this antibody had very little background in somatic (follicle) nuclei in the *Drosophila* ovary (Supporting Information, Figure S1) and was highly specific to DSBs, this antibody can be readily used in immunocytological assays to detect the rapid response to both pre-programmed DSBs and DSBs created during endocycle S phase.

### Specificity of γ-H2AV antibody in Western blots to DSBs created by IR

**Figure 3:**
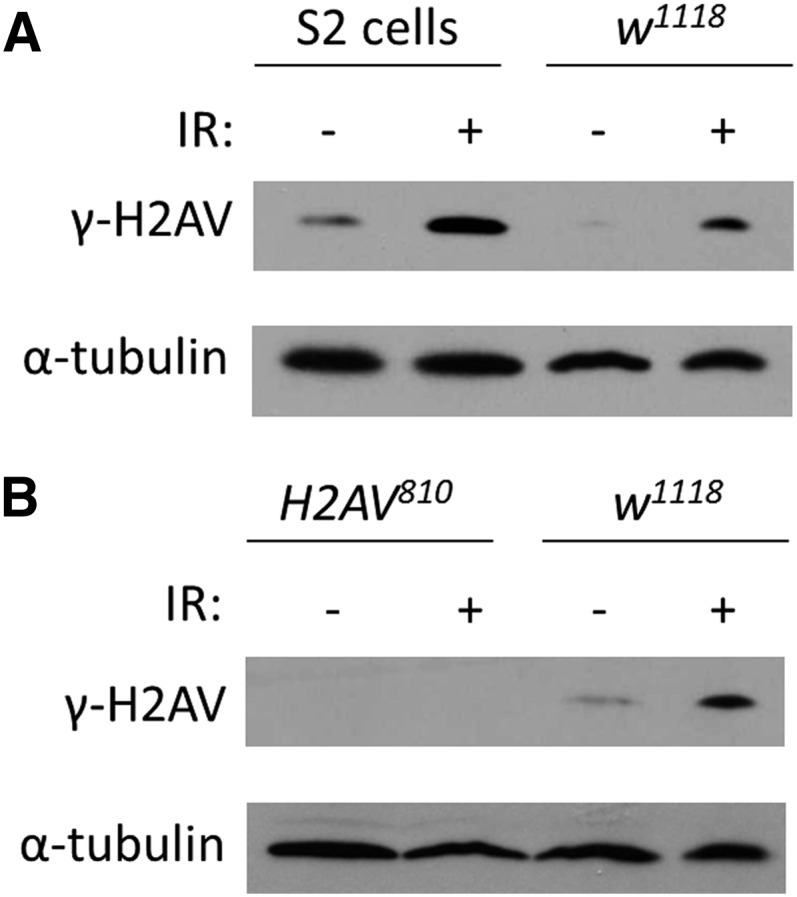
Monoclonal antibody γ-H2AV binds phosphorylated H2AV as assayed by Western blot. (A) Western blot of extracts from S2 cells and wild-type flies with and without IR treatment. (B) Western blot of amorphic *H2AV^810^* allele and wild-type larvae with and without IR.

We tested the specificity of the monoclonal γ-H2AV antibody in Western blot on *Drosophila* S2 cells, whole flies, and larvae with and without IR. IR is known to cause DSBs that are rapidly modified at the DSB site through the phosphorylation of the H2A variant (Rogakou *et al.* 1998). We therefore analyzed both S2 cells and whole flies that were exposed to IR to control untreated tissue. Within 10 min for S2 cells and 5 min for whole flies, we found a dramatic increase in the amount of γ-H2AV as assayed by Western blot (Figure 3A), indicating that the antibody recognized the increase in H2AV phosphorylation resulting from DSBs created by IR.

To determine where the antibody bound nonspecifically to other histone modifications, we analyzed the ability to detect any signal in *H2AV^810^*, a mutation that results in a failure to make H2AV (van Daal and Elgin 1992) and compared with to wild-type tissue. The *H2AV^810^* mutation is lethal in 3^rd^ instar larval stage so 2^nd^ instar larval tissue was used for both *H2AV^810^* and control tissues. After exposure to IR, we were only able to detect γ-H2AV in wild-type larvae by Western blot (Figure 3B), indicating that the antibody is specific to the phosphorylated form of H2AV.

### Specificity of γ-H2AV antibody to DSBs created by IR in an immunocytological assay

**Figure 4:**
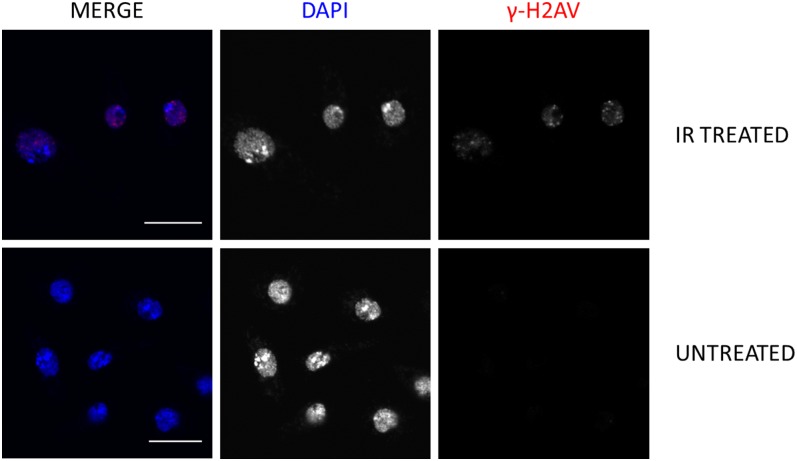
Staining of γ-H2AV in response to IR in S2 cells. Characteristic γ-H2AV foci are present 15 min after S2 cells are exposed to 1000 rads of IR. No γ-H2AV staining is observed in the untreated group. Protein A−purified mouse γ-H2AV (red) and DAPI (blue). Scale bar: 10 µm.

In addition to being able to detect DSBs created by IR in Western blot, we also tested the ability of the antibody to detect these DSBs in S2 cells using an immunocytological assay. As shown in Figure 4, only after treatment of S2 cells to IR were we able to visualize the characteristic γ-H2AV foci with the γ-H2AV antibody. This indicates the antibody will be useful for detecting DSBs created by IR in both Western blots and immunocytological assays.

### Future applications for antibody

As we have shown in this study, the monoclonal antibody created to γ-H2AV specifically recognizes the phosphorylation of H2AV that results from programmed DSBs in meiotic tissue, DSBs created by endocycle S phase and those DSBs formed by gamma radiation in whole tissue and in *Drosophila* cell culture in both Western blot and immunocytological assay. As the first highly specific, monoclonal antibody made to the *Drosophila* γ-H2AV modification, it will be a useful tool to many in the *Drosophila* community. It may now be possible to analyze, at high-resolution, genome-wide localization of γ-H2AV by chromatin immunoprecipitation and chromatin immunoprecipitation sequencing.
